# The Influence of Oat β-Glucans of Different Molar Mass on the Properties of Gluten-Free Bread

**DOI:** 10.3390/molecules29194579

**Published:** 2024-09-26

**Authors:** Angelika Bieniek, Krzysztof Buksa

**Affiliations:** Department of Carbohydrate Technology and Cereal Processing, University of Agriculture in Krakow, Balicka 122, 30-149 Krakow, Poland; angelika.bieniek@student.urk.edu.pl

**Keywords:** oat β-glucans, gluten-free bread, molar mass

## Abstract

The influence of β-glucans on the properties of gluten-free dough and bread is still not fully explained, with the literature suggesting both positive and negative effects. The aim of this study was to investigate the effect of the molar mass of oat β-glucans on the properties of gluten-free bread. Gluten-free breads were baked under standardized conditions from a model gluten-free mix without and with a 1% or 2% share of oat β-glucans of a low molar mass of 24,540 g/mol, a medium molar mass of 85,940 g/mol and a high molar mass of 1,714,770 g/mol. The share of β-glucans affected the increase in water addition to the baking mix and dough yield proportionally to the molar mass and amount of β-glucans. The β-glucans of the highest molar mass, particularly at a 2% share, were most effective in increasing bread volume, reducing hardness and increasing the moisture content of the bread crumb on the day of baking, as well as reducing the increase in hardness and maintaining a high moisture content of the bread crumb after 1 day of storage, compared to bread without added β-glucans.

## 1. Introduction

Unlike bread doughs containing gluten, gluten-free dough lacks a three-dimensional protein–starch network, which results in an inelastic, difficult-to-process dough that affects the final and overall quality of the bread [1]. β-glucans belong to the soluble fraction of dietary fiber, which is found in high amounts primarily in cereal grains, but also in fungi (including yeast) and algae [2]. Cereal β-glucans consist of long, linear chains composed of glucose residues linked by β-(1→4) and β-(1→3) glycosidic bonds [2]. The content of β-glucans in oat grain is 2.1–6.1% [3,4,5,6,7]. β-glucans are abundant in the outer parts of the grain, so their content is particularly high in oat bran [4,7,8,9]. β-glucans are most commonly extracted from oat and barley grains with yields in the range of 1.82–16.5% [10,11].

Based on the literature data, the average molar mass of oat grain β-glucans ranges widely from 35,000 g/mol to 2,300,000 g/mol [4,12,13,14,15,16,17,18]. The large variation in the molar mass of oat β-glucans is due to the diversity of the raw material [13,15,19,20,21], the solvent used [18], the extraction conditions [22] and the methodology applied to determine these values [2,15].

Using an appropriate addition of β-glucans, the properties of bread can be improved, especially when flour of weak baking quality was used in its production [23,24]. A previous study showed that the addition of water to the dough increased proportionally to the share of β-glucans in the gluten-free baking mix [23]. The consequence of increased water addition to flour is a higher yield of wheat and gluten-free dough and bread. Kurek et al. [25] showed that as the share of oat β-glucans increases, the yield of wheat dough increases. The share of oat β-glucans may also affect the increased baking loss of gluten-free bread, compared to bread without added β-glucans [23]. In addition, it has been shown that the higher the share of β-glucans in gluten-free bread, the higher the moisture content of its crumb, compared to bread without added β-glucans [23,24].

According to the literature, the effect of the share of β-glucans on the volume of gluten-free bread is ambiguous. The share of β-glucans increased the volume [26,27], had no significant impact on [28] or caused a decrease in the volume [23,27,28] of gluten-free breads, compared to breads without added β-glucans. The effect of β-glucans on bread crumb hardness is also inconclusive. The share of oat β-glucans had no impact on [23,27], caused an increase in [23] or caused a decrease in the hardness of the crumb of gluten-free bread, compared to bread without added β-glucans. The ambiguous, differential influence of β-glucans on the volume and texture of gluten-free bread may have resulted from the use of different amounts of β-glucans and differences in the structure and molar mass of the β-glucans. In addition, other differences may include the use of different raw materials and different recipes for baking gluten-free breads.

The aging of gluten-free bread continues to pose problems for the baking industry. Gluten-free bread ages quickly, mainly due to particularly intense starch retrogradation [29]. Previous publications have shown that the share of hydrocolloids, particularly β-glucans, can delay aging and maintain the high quality of gluten-free bread during storage [24,26,30].

In addition, it was found that the high water-binding capacity of β-glucans and the increased addition of water to the dough have been reported to probably reduce water loss during storage and decrease the rate of staling of gluten-free bread [24]. Previous research has shown that β-glucans can also affect the migration of water from the center of the crumb to the crust of the bread during storage [31].

The effect of the molar mass of β-glucans on the properties of gluten-free bread is unclear. Previous research found a higher reduction in the volume and an increase in the hardness of the crumb of gluten-free bread when β-glucans of a molar mass of 83,000 g/mol were used than those of a molar mass of 192,000 g/mol [27]. On the other hand, the share of oat β-glucans of a molar mass of 650,000 g/mol did not significantly affect the volume of the gluten-free bread, but reduced the hardness of its crumb, compared to bread with a share of β-glucan of lower molar mass [27]. It should be noted that the effect of the molar mass of β-glucans on the quality of dough and gluten-free bread was mainly studied using commercial preparations of β-glucans, often with low or undefined purity, which involved the simultaneous addition of other substances to the dough, such as proteins and dextrins, which could mask the benefits of β-glucans [27]. In addition, the reason for the inconclusive effect of the molar mass of β-glucans on the properties of gluten-free bread may have been due to the interaction of β-glucan molecules with numerous gluten-free dough ingredients.

So far, there has been no research on the impact of β-glucans and their molar mass on the properties of gluten-free bread baked under standardized conditions using the direct method. The aim of this study was to investigate the influence of the molar mass of oat β-glucans, native and modified (partly hydrolyzed), on the properties of model gluten-free bread. For this purpose, β-glucans of low, medium and high molar mass were produced and applied in the baking of gluten-free bread. To limit the interactions of various compounds, the gluten-free breads were baked from a model mix consisting only of basic ingredients.

## 2. Results and Discussion

To study the impact of the molar mass of β-glucans on the properties of gluten-free bread, it was necessary to obtain β-glucans that differed significantly in molar mass. Based on information from the literature and preliminary studies, water extraction at room temperature was used to obtain β-glucan of medium molar mass (BG-MMW preparation). To obtain β-glucan of low molar mass (BG-LMW preparation), part of the preparation of β-glucan isolated at room temperature (BG-MMW) was dissolved, subjected to partial enzymatic hydrolysis by lichenase and then dried. To obtain high-molar-mass β-glucan (BG-HMW formulation), the precipitate remaining after β-glucan extraction at room temperature (BG-MMW) was re-extracted at 95 °C.

Table 1 shows the chemical composition and properties of β-glucan preparations extracted from whole-grain oat flour. The yield obtained for the BG-MMW preparation was lower compared to the yields reported in the literature, which range from 1.82% to 7.63% [32,33]. The lower yield of BG-MMW can be explained by the different extraction conditions, specifically the use of room-temperature water. According to the literature [2,6,18,22], using hot water or alkaline solutions results in a higher yield of β-glucans. The yield of BG-HMW was even lower than that of BG-MMW, because a significant part of the β-glucans had already been extracted from the material during the room-temperature extraction of BG-MMW.

All β-glucan preparations were characterized by a very similar content of total sugars and composition of sugar residues released after acid hydrolysis, and the dominant sugar was glucose, which was the monomer of β-glucan molecules (Table 1). The β-glucan content of the preparations was about 74%, which was consistent with the results of other studies, where oat β-glucan preparations had β-glucan content ranging from 74% to 97% [13,14,15,20]. The arabinoxylan (AX) content in the β-glucan preparations was about 6%, which was consistent with data from other research, where β-glucan preparations from oat grains had AX contents ranging from 5.5% to 14.1% [34]. The resulting galactose and mannose content in the preparations (Table 1) may have originated from small amounts of polysaccharides, such as galactomannans, which naturally occur in cereal grains in small quantities and may have been extracted along with the β-glucan [35,36].

The protein content of β-glucan preparations (Table 1) was about 7% and can be considered typical, because the study by Skendi et al. [15] reported that the protein content of β-glucan preparations from oat grain ranged from 3.1% to 9%. The β-glucan preparations did not contain starch, which was confirmed by an iodine test and which indicates that the amylase treatment during extraction was carried out correctly.

**Figure 1:**
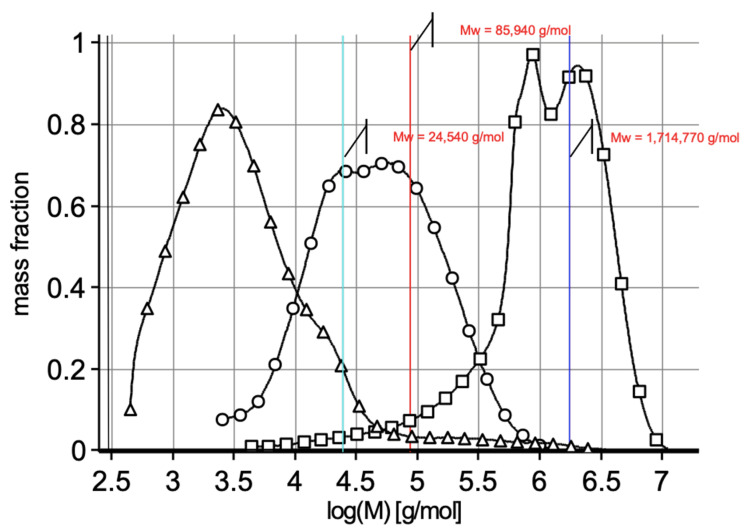
Molecular mass distribution profiles of low (-Δ-)-, medium (-○-)- and high (-□-)-molar-mass β-glucan.

Figure 1 shows the molar mass distribution profiles of low (BG-LMW)-, medium (BG-MMW)- and high (BG-HMW)-molar-mass β-glucan.

Based on the molar mass distributions (Figure 1), the molecular parameters of β-glucans were determined, including the weight-average molar mass (M_w_), number-average molar mass (M_n_) and dispersity (Ð). The molar mass of the room-temperature water-extracted β-glucan (BG-MMW), with an average molar mass of 85,940 g/mol, was similar to the data presented in the literature, where this parameter ranged from 35,000 g/mol to 250,000 g/mol [13,14]. The partially hydrolyzed β-glucan (BG-LMW, low molar mass) had the lowest molar mass of 24,540 g/mol, which was lower than the value reported by Tosh et al. [37], where the molar mass of hydrolyzed oat β-glucan was 30,800 g/mol. β-glucan in the hot-water-extracted preparation (BG-HMW, high molar mass) had the highest molar mass, and this value was lower than the highest molar mass of oat β-glucan (2,179,700 g/mol) reported in the literature data [38]. The differences in the molar mass of β-glucans from oat grain were probably caused by the different types of raw material, different extraction conditions and different methods of determining the molar mass.

To study the impact of the molar mass of β-glucans on the properties of gluten-free bread, it is necessary to use standardized (identical) conditions for dough preparation and baking gluten-free bread without (control) and with a share of β-glucans. To achieve this, the optimal composition of the model gluten-free mixture was developed in preliminary studie, helping to eliminate as many factors as possible that could interfere with the interpretation of the obtained results. The model baking mix (gluten-free flour) was composed of rice flour, corn starch and sucrose. The addition of other ingredients, especially hydrocolloids, would not fully allow us to determine the effect of the molar mass of β-glucans on the properties of gluten-free dough and bread.

Water was added to the model gluten-free mixtures to achieve a dough firmness in the range of 0.4–0.5 N, as determined by a backward extrusion test using a texturometer. The addition of water and the dough yield of the model gluten-free dough without and with a share of oat β-glucan preparations are shown in Table 2.

The level of water addition to the model gluten-free mixture without added β-glucans was comparable to the literature data [39].

The preparation containing β-glucan of the highest molar mass (BG-HMW) had the greatest impact on the amount of water added to the gluten-free mix, compared to the mix without added β-glucans (Table 2). The 2% share of the BG-HMW preparation resulted in a 24% increase in the addition of water to the gluten-free mixture. A similar effect of β-glucans on the addition of water to the dough from the gluten-free mix was observed by Ronda et al. [23]. Compared to the control sample, the preparation of β-glucan of the lowest molar mass (BG-LMW) led to the lowest increase in the amount of water added to the model gluten-free dough. At a 1% share, it did not significantly (*p* < 0.05) affect this parameter. The share of unmodified β-glucan (BG-MMW) of medium molar mass, at both levels of substitution, caused an increase of 5% and 9%, respectively, in water addition to the gluten-free model mix, compared to the baking mix without added β-glucans.

The increase in the level of water addition to the model gluten-free mixture was proportional to the preparation’s share, which was consistent with information presented in the literature, where the addition of water increased with the share of β-glucans in the gluten-free baking mix [23].

Dough yield is correlated with the level of water addition to the baking mix. The yield of the dough constituting the control sample (without the addition of β-glucans) was similar to the yield of this type of dough presented in the literature [40]. The 2% share of BG-HMW increased dough yield to the greatest extent, by 21.3%, compared to dough without added β-glucans, while the 1% share of BG-LMW had the lowest impact. The share of BG-MMW β-glucan of a molar mass of 85,940 g/mol increased the yield of gluten-free dough by 8%. Similar to the addition of water, the yield of gluten-free dough increased with the share of β-glucan preparations.

To eliminate the influence of fermentation and baking conditions on bread properties, the fermentation and baking of all dough pieces were carried out simultaneously under the same standardized conditions. Table 3 shows the properties of gluten-free bread without and with the share of β-glucans.

The baking loss (BL) of bread expresses the difference between the weight of the formed dough piece and the weight of the bread immediately after removal from the oven. The baking loss of gluten-free bread without the share of β-glucans was about 15%. Total baking loss (TBL) includes the difference between the weight of the formed dough piece and the weight of the cooled bread. The total baking loss of the gluten-free bread without the addition of β-glucans was higher than in the study of Ronda et al. [23]. The observed difference may have been due to the fact that in the study by Ronda et al. [23], the baking mix included other ingredients like hydroxypropylmethylcellulose, which is a hydrocolloid, and these compounds have the ability to bind and retain water.

The share of β-glucans, regardless of their molar mass, did not significantly (*p* < 0.05) affect the baking loss of gluten-free bread (BL). The preparation BG-MMW, especially in the amount of 2%, increased the total baking loss (TBL) of gluten-free bread by about 2.8%, compared to bread without added β-glucans [23]. An effect of β-glucan of a similar molar mass of 472,000 g/mol on the higher baking loss of gluten-free breads was observed by Ronda et al. [23]. The share of β-glucan of the highest molar mass (BG-HMW) did not significantly (*p* < 0.05) affect the total baking loss of gluten-free bread compared to bread without added β-glucans. Pastuszka et al. [28] showed that the share of oat β-glucan did not affect the total baking loss of gluten-free bread. The observed differences can be explained by the impact of β-glucan not only on the addition of water to the dough, but also on the volume of the bread, as during baking and cooling, water evaporates to a higher degree from bread of a high volume due to a larger evaporation surface [23].

The volume of bread from a given amount of dough (BV) allows us to characterize the influence of dough ingredients on the strength of the bread crumb structure [41]. The volume of gluten-free bread (Table 3, BV) without the addition of β-glucans (control sample) was generally smaller, with reference to the literature data [26], which could be due to the limited composition of the model gluten-free mix used. The use of a 1% share of β-glucans resulted in a significant increase in bread volume (by approximately 8%) compared to the control sample baked without the addition of β-glucans. At a 1% share, all preparations (BG-LMW, BG-MMW, BG-HMW) increased the bread volume. Using a similar share of oat β-glucans, Pastuszka et al. [28] did not observe the effect of β-glucans on bread volume, which could be due to the more complex composition of the gluten-free mixture and possible interactions of added β-glucans with dough ingredients, hindering the interpretation of the influence of β-glucans. The use of a 2% share of BG-LMW was also as effective as the use of a 1% share. The use of a 2% share of BG-HMW and BG-MMW had no influence on bread volume, compared to the control sample.

The specific bread volume (SBV) is the volume of bread baked from 100 g of flour, which includes dough yield. The SBV of gluten-free bread baked from a model gluten-free mix without the addition of β-glucans was similar to data presented in the literature [23]. The use of a 2% preparation of high-molar-mass β-glucan (BG-HMW) was most effective in increasing the specific volume of gluten-free bread, which was consistent with data presented in the literature [27]. The use of a 1% share of each of the three preparations tested also considerably affected SBV. In contrast, no significant (*p* < 0.05) effect on the specific volume of model gluten-free bread was observed when a 2% share of the medium molar mass (BG-MMW) β-glucan preparation was applied, compared to bread without added β-glucans (Table 3). It should be noted that the share of low-molar-mass β-glucan (BG-LMW) in the amount of 1% and 2% had a positive impact on increasing the specific volume of gluten-free bread, which was consistent with data in the literature [27].

Table 4 shows the results of the determined texture parameters—the hardness and adhesiveness of the crumb of gluten-free breads without and with the share of β-glucans determined on the day of baking and after 1 day of storage.

The texture parameter most often measured when testing the textural properties of bread crumb is its hardness. On the day of baking, the hardness of the crumb of gluten-free bread without added β-glucans (Table 4) was higher than that reported in the study by Ronda et al. [23]. The application of the share of all types of β-glucan preparations (Table 4) resulted in a decrease in the hardness of the crumb of gluten-free breads, compared to breads without added β-glucans. The 2% share of the BG-HMW preparation had the highest influence on the reduction (by more than 69%) of the crumb hardness of model gluten-free bread. A similar reduction in bread crumb hardness was reported by Pérez-Quirce et al. [27] using a 1.3% share of oat β-glucan of a molar mass of 650,000 g/mol in gluten-free bread [27]. The 1% and 2% shares of the low-molar-mass β-glucan (BG-LMW), as well as the 1% share of the medium-molar-mass β-glucan (BG-MMW), reduced the hardness of the crumb of gluten-free bread by 43.4%, 46.9% and 38.5%, respectively, compared to bread without added β-glucans, which also confirms data presented in the study of Pérez-Quirce et al. [27]. Only a 2% share of the BG-MMW preparation did not significantly (*p* < 0.05) affect the hardness of the crumb of the gluten-free bread, compared to the bread without added β-glucans, which was associated with a particularly low bread volume. 

The determined adhesiveness of the crumb of gluten-free bread without the addition of β-glucans (Table 4) was lower, compared to literature data [42], which can be explained by the limited composition of the model gluten-free mix used for bread baking. There was no significant impact (*p* < 0.05) of both the share and molar mass of β-glucans on the adhesiveness of the crumb of model gluten-free bread, compared to bread without added β-glucans.

One of the ways to determine the degree of aging of bread is to determine changes in the hardness of the bread crumb on the day of baking and during storage. Table 4 shows the results of the hardness and adhesiveness of the crumb of gluten-free breads without and with the share of β-glucans on the day of baking and after 1 day of storage.

After 1 day of storage, the hardness of the crumb of gluten-free bread without added β-glucans increased more than 2 times, compared to the hardness of the bread on the day of baking (Table 4). The observed changes confirm the results of Ronda et al. [23], which also showed that the hardness of the crumb of gluten-free bread without added β-glucans, after 1 day of storage, was significantly higher than on the day of baking [23].

The use of all types of preparations, at both levels of substitution, resulted in a reduction in the increase in crumb hardness during storage and in the hardness of the bread crumb after 1 day of storage, compared to the crumb of the bread constituting the control sample. The share of the BG-HMW preparation, especially in the amount of 2%, effectively reduced the increase in the hardness of the bread crumb after 1 day of storage, confirming the results of a study by Ronda et al. [23], according to which β-glucans influenced the retrogradation of starch by interfering with the associations between amylopectin molecules [23]. The 2% share of the medium-molar-mass β-glucan preparation (BG-MMW) had the least effect on reducing the increase in the hardness of the crumb of gluten-free bread after 1 day of storage, compared to the hardness of the bread on the day of baking.

Gluten-free bread made from blends of starches of various botanical origins is crumbling and inflexible. High crumb adhesiveness is not desirable, as such bread is hard to cut. After 1 day of storage, the crumb adhesiveness of gluten-free bread without added β-glucans increased to the highest extent, compared to gluten-free breads with added β-glucans. After 1 day of storage, the 2% share of the BG-HMW preparation had the best effect on reducing the adhesiveness of the bread crumb, compared to the bread on the day of baking. The share of the other β-glucan preparations was less effective in limiting the increase in crumb adhesiveness during storage.

To study the influence of β-glucans on the water content of the bread crumb, as well as the migration of water in the bread crumb on the day of baking and after 1 day of storage, the moisture content of the bread crumb in the central and peripheral parts (near the crust) was determined, and Table 5 shows the results of the determinations.

According to the statistical analysis (Supplementary Table S1), a significant effect of β-glucan type (molar mass), β-glucan share and storage time on bread crumb moisture in the central and peripheral parts was observed. The bread crumb moisture content of gluten-free bread without added β-glucans determined in the middle part (Table 5) was similar to data in the literature [28]. The use of all preparations, at both substitution levels, resulted in bread with the desired higher crumb moisture content. The bread crumb moisture content was especially dependent on the molar mass of β-glucans (Table 5 and Supplementary Table S2) which was a consequence of the water-binding capacity of these hydrocolloids. In both the central and peripheral parts of the crumb, the preparation of β-glucan of the highest molar mass (BG-HMW), at both levels of substitution, was most effective in increasing the moisture content of the crumb of gluten-free bread, compared to bread without added β-glucans (Table 5), and the value of this parameter increased by 5.5% and 9.3% (central part) and by 6.2% and 8.4% (peripheral part), respectively. This was due to the highest amount of water added to the dough, as also indicated by the study conducted by Ronda et al. [23]. On the other hand, the highest addition of water to the dough was due to the highest molar mass of β-glucans. The second factor responsible for bread crumb moisture was the share of β-glucans; however, this factor seems to play a less important role in shaping the discussed parameter (Supplementary Table S1), compared to the molar mass of β-glucans. It was noted that the higher the share of β-glucans in the model gluten-free bread, the higher the moisture content of its crumb, which was consistent with data in the literature [23].

The statistical analysis of the crumb moisture content of gluten-free breads without and with β-glucans showed that after 1 day of storage, the moisture content of the middle part in most cases did not change significantly (*p* < 0.05), compared to the crumb moisture content of breads on the day of baking (Table 5 and Supplementary Table S2). According to the results presented in Table 5, only in the crumb of breads baked without β-glucan addition and with a 2% share of BG-HMW after 1 day of storage was there significantly (*p* < 0.05) lower moisture content, compared to the value of this parameter on the day of baking.

After 1 day of storage, the moisture content of the peripheral part of gluten-free bread without the addition of β-glucans (control sample, Table 5) decreased by 2.1%, compared to the moisture content of the bread crumb on the day of baking. Only when a 1% share of the BG-MMW preparation was used did the crumb moisture of the peripheral part of gluten-free bread not change after 1 day of storage, compared to bread with the same β-glucan preparation on the day of baking. In the crumb of breads with 1% and 2% shares of BG-LMW and BG-HMW and with a 2% share of BG-MMW, drying of the peripheral parts of the crumb was observed (Table 5 and Supplementary Table S2).

According to the results presented in Table 5, a tendency was observed for BG-MMW, especially at a 1% share, to minimize the changes in the moisture content during 1 day of storage time.

In summary, both the share and, in particular, the molar mass of β-glucans have a significant effect on water addition to the dough (analysis of variance, Supplementary Table S3) and, as a consequence, other water-related parameters, i.e., dough yield and bread crumb moisture (determined both in the central and peripheral parts of the bread crumb). Moreover, the analysis of variance (Supplementary Table S3) showed that the effect of β-glucan type (molar mass) was dependent on β-glucan share (significant interactions of molar mass of β-glucans and β-glucan share). This resulted from the high water-binding capacity of high-molar-mass β-glucan [43]. Only in the case of the moisture of the crumb in the peripheral part of breads, due to the uneven evaporation of water from the outer parts of the bread crumb during the baking and cooling process and water migration in the bread crumb, was the effect of the molar mass of β-glucans factor independent of the β-glucan share factor (interaction not significant, Supplementary Table S3). β-glucan share strengthened the bread crumb structure and particularly affected bread volume (BV) and the water evaporation surface, which is related to the bread volume. The impact of the β-glucan share and molar mass of β-glucan on BL was not significant; however, after cooling, a longer duration of water evaporation from the bread revealed the impact of β-glucan type (molar mass), and an interaction of the molar mass of β-glucan factor and the β-glucan share factor on total baking loss (TBL) was observed. The specific bread volume (SBV) was more dependent on the molar mass of the β-glucan used, which resulted from a strong impact of the molar mass of β-glucans on water addition and dough yield [43]. The effects of the share of β-glucans and the interaction of the molar mass of β-glucan factor and the β-glucan share factor were also significant (Supplementary Table S3). The molar mass of β-glucans also strongly influenced breadcrumb hardness. In the case of breadcrumb hardness, the analysis of variance showed that the effect of the molar mass of β-glucan factor was dependent on the β-glucan share factor (significant interaction of molar mass of β-glucans and β-glucan share). This was a consequence of water’s binding capacity and ability to strengthen the structure of the bread crumb, especially observed in the case of the use of high-molar-mass β-glucan. It could be concluded that the appropriate amount of water bound by non-starch polysaccharides affects the stability of both gas pores in dough and dough structure, resulting in a higher volume and lower hardness of bread. However, the mechanisms behind this process are still not well explained [43]. The significant impact of the β-glucan share and molar mass of β-glucans on bread adhesiveness was revealed after one day of storage (not significant on the day of baking), and the effect of the molar mass of β-glucans factor was not dependent on the β-glucan share factor.

The baking of model gluten-free bread under standardized conditions with the share of β-glucans of different molar masses allowed the selection of the type and amount of preparation that most effectively improved the technological properties and quality characteristics of the bread. On the basis of the results obtained, the preparation of β-glucan of a high (1,714,770 g/mol) molar mass (BG-HMW), in the amount of 2%, can be recommended for the baking of gluten-free bread due to its highest impact on improving the technological properties, i.e., the addition of water to the baking mix and the associated dough yield, as well as the quality characteristics, i.e., increasing the volume, reducing the hardness and increasing the moisture content of the crumb of gluten-free bread.

## 3. Materials and Methods

The material for the isolation of β-glucans was BIO oat flour type 2000 (GRANO, Kalisz, Poland) with a composition of 5.9/100 g fat, 65 g/100 g carbohydrates, 7.7 g/100 g fiber and 9.4 g/100 g protein. The model gluten-free baking mixture was prepared from rice flour (Bezgluten, Koniusza, Poland), corn starch (Bezgluten, Koniusza, Poland) and sucrose (Südzucker, Wroclaw, Poland). Other baking additives included freeze-dried *Saccharomyces cerevisiae* yeast from Lesaffre, France, and refined, non-iodized salt (NaCl, Avantor Performance Materials Poland S.A., Gliwice, Poland).

### 3.1. Isolation and Modification of Oat β-Glucans

#### 3.1.1. Isolation of Oat β-Glucans

To obtain β-glucans of different molar masses, a new method for the isolation and modification of β-glucans was developed. β-glucans were isolated from 1 kg of oat flour. For a single batch of the isolation of β-glucans from oat flour, 200 g of flour was taken and mixed with 720 mL of ethanol and 80 mL of deionized water, and the whole mixture was heated for 2 h at 70–90 °C with periodic stirring. After cooling, the mixture was centrifuged, and the resulting sediment was left to dry. In the next step, water extraction was carried out. For this purpose, water was poured on the sediment (in a ratio of 1:20), stirred for 2 h at room temperature (25 °C) and then centrifuged. After centrifugation, the supernatant was collected, and to the precipitate, a new portion of water was added (in a ratio of 1:20), intensively stirred for 3–4 h at 95 °C, cooled and then centrifuged. After centrifugation the supernatant was collected. To the resulting supernatants (from both extractions at room temperature and 95 °C), equilibrated at 37 °C, α-amylases solution (6000 U of α-amylases from porcine pancreas and *Aspergillus oryzae*; Sigma Aldrich, Saint Louis, MI, USA) was added and the whole solution was incubated at 37 °C until a negative iodine test result was obtained, after which the mixture was boiled for 5 min to inactivate the enzymes. The cooled solutions were purified with 4 volumes of ethanol to precipitate β-glucan, and then recovered by centrifugation. The resulting sediment was treated with acetone and centrifuged. This step was performed two more times, and the precipitated β-glucans were dried for 24 h, at 40 °C. The preparation of β-glucan after extraction at room temperature was denoted as medium-molar-mass β-glucan (BG-MMW) and the preparation of β-glucan after extraction at 95 °C was denoted as high-molar-mass β-glucan (BG-HMW). The resulting preparations were weighed to determine the yield of β-glucan.

#### 3.1.2. Modification of Oat β-Glucan by Partial Enzymatic Hydrolysis

In order to obtain low-molar-mass β-glucan, 9 g of the medium-molar-mass β-glucan (BG-MMW) was solubilized in 50 mL of buffer and partially hydrolyzed using 20 µL lichenase at 35 °C for 2 h, after which the mixture was boiled for 5 min to inactivate the enzyme. The cooled solutions were purified with ethanol, followed by acetone, as described in Section 3.1.1. The precipitated β-glucan preparation was dried for 24 h at 40 °C and denoted as low-molar-mass β-glucan (BG-LMW).

### 3.2. Determination of the Monosaccharide Composition of β-Glucans

The monosaccharide composition of β-glucan preparations was determined using the HPLC/RI method after acid hydrolysis according to Buksa et al. [44].

### 3.3. Determination of Molecular Properties of β-Glucans

The distribution of the molar mass of β-glucans was evaluated using HPSEC/RI according to Buksa et al. [45]. The chromatographic system consisted of a Knauer chromatograph (Knauer, Berlin, Germany), equipped with a combination of OHpak SB-806HQ and SB-804HQ (Shodex, Tokio, Japan) columns and a refractometric detector (Knauer, Berlin, Germany). Separation was performed at a column temperature of 60 °C. We used 0.1M NaNO_3_ at a flow rate 0.6 mL/min as an eluent. The calibration of the HPSEC system was performed with pullulan standards (Shodex Standard, Tokio, Japan; Macherey—Nagel, Dueren, Germany) with known molar masses and glucose (Sigma-Aldrich, Saint Louis, MI, USA). The molar mass distribution and apparent average molar mass Mw (related to pullulan standards) were calculated using the software programs Eurochrom (Knauer, Berlin, Germany) and Clarity (ver. 4.0.1.700, DataApex, Prague, Czech Republic).

### 3.4. Preparation of Dough without and with a Share of β-Glucans and Baking of Model Gluten-Free Bread by Direct Method

The model dough was made from a baking mixture (model gluten-free mix) composed of 78.4% rice flour, 19.6% corn flour and 2% sugar. According to the basic recipe, for the dough prepared from 100 g of gluten-free mixture, water (measured by a back extrusion test), 1.8% salt and 3.5% yeast were used. The addition of water was adjusted to ensure an optimal dough firmness of 0.4–0.5 N, which was measured on a TA.XT Plus texture analyzer equipped with a back extrusion rig (Stable Microsystems, Godalming, UK) by a back extrusion test at a test speed of 0.83 mm/s according to Korus et al. [46]. The mentioned recipe was used to make the dough and bread, constituting the control sample. In making the other doughs, β-glucan preparations of 1% and 2% by weight of model mix (flour) were added in place of flour. The dough was hand-mixed for 12 min, and then, 60 g of dough was transferred into molds and placed in a fermentation chamber. At least six breads were baked under standardized conditions in one batch for each bread variant (three breads were analyzed on the day of baking and three after 1 day of storage) and all variants were baked together. Fermentation was carried out at 30 °C and 80% humidity for 23 min, after which the dough was baked at 230 °C for 20 min in a Miwe Condo modular electric oven, type C-52. After putting the breads in the oven, the baking chamber was steamed once. The breads were cooled for 1 h at room temperature (about 25 °C). The breads were stored in foil bags in a chamber for 24 h at 25 °C.

#### 3.4.1. Baking Loss

Baking loss was determined according to Murat Karaoğlu et al. [47]. The resulting breads were weighed hot (immediately after removal from the oven), cooled and weighed again. Based on the difference in the weights of the dough pieces before baking and the weight of the bread after baking, baking loss (BL) was calculated (the percentage of water that had evaporated from the bread during baking). Meanwhile, based on the difference in the weights of the dough pieces before baking and the weight of the bread after baking and cooling total baking loss (TBL) was determined (the percentage of water that had evaporated during baking and cooling after baking).

#### 3.4.2. Volume

After cooling, the volume of the baked breads (BV) was determined by a Volscan Profiler 3-dimensional laser-based scanner (Stable Microsystems, Godalming, UK), according to the manufacturer’s manual. The specific volume of the bread (SBV) was determined by converting the bread volume (BV) to the volume of bread obtained from 100 g of flour.

#### 3.4.3. Moisture

The moisture content of the crumb on the day of baking and after 1 day of storage was determined by drying for 1 h at 130 °C, according to AOAC 925.10 [48]. To determine the moisture content of the crumb, samples were taken from the central part of the bread and from the peripheral part, which was very close to the crust.

#### 3.4.4. Texture

Texture parameters were determined on the day of baking and after 1 day of storage using the TA.XT Plus texture analyzer (Stable Microsystems, Godalming, UK) according to the standard program [49]. A sample of bread crumb, taken from the base of the loaf with a height of 30 mm, was pressed to reach 10 mm maximum strain by a P/20 aluminum compression platen with a diameter of 15 mm, in two cycles with a 5 s delay. From the resulting parameters of TPA (Texture Profile Analysis), only the hardness and adhesiveness of the crumb were used as indicators of its textural properties. The calculations were performed using the attached software, Texture Exponent (ver. 3.0.5.0, Stable Microsystems, Godalming, UK).

#### 3.4.5. Statistical Analysis

All experiments or tests were performed at least in triplicate. A statistical analysis (Statistica v. 9.0 software, StatSoft, Inc., Tulsa, OK, USA) of variance (one-way ANOVA, Tukey’s test at significance level 0.05; the data are shown within the manuscript) was performed. Two-way ANOVA (Supplementary Materials) was performed to study the effect of each variable (molar mass of β-glucan, β-glucan share, day of storage, part of the bread crumb) as well as any interactions between them.

## 4. Conclusions

The share of β-glucans increased water addition to the baking mix and dough yield proportionally to the amount and molar mass of β-glucans. The 2% share of the high-molar-mass β-glucan preparation (BG-HMW) resulted in the highest increase in water addition to the model gluten-free baking mix and dough yield (by about 20%), compared to dough without β-glucan addition.

A 2% share of the BG-HMW preparation had the best effect on increasing the volume and decreasing the hardness of the crumb of gluten-free bread. The 1% and 2% shares of the low-molar-mass β-glucan preparation (BG-LMW), as well as the 1% share of the medium-molar-mass β-glucan (BG-MMW), had a positive influence on the volume and on reducing the hardness of the crumb of gluten-free bread (by 43.4%, 46.9% and 38.5%, respectively), compared to bread without added β-glucans.

The preparation of β-glucan with the highest molar mass (BG-HMW), at both levels of substitution, was most effective in increasing the moisture content of the crumb of gluten-free bread, in the central and peripheral parts of the crumb, compared to bread without added β-glucans.

The share of BG-HMW preparation, especially in the amount of 2%, was most effective in maintaining the low hardness and high moisture content of the crumb of model gluten-free bread after 1 day of storage.

## Figures and Tables

**Table 1:** Chemical composition and properties of β-glucans obtained from whole-grain oat flour.

Component	BG-LMW *	BG-MMW *	BG-HMW *
Yield [%]	nd	1.65 ± 0.37 b	0.33 ± 0.09 a
Glucose [%]	81.4 ± 1.9 a	80.3 ± 1.7 a	84.2 ± 2.2 a
Xylose [%]	2.9 ± 0.4 a	2.7 ± 0.8 a	2.1 ± 0.5 a
Galactose [%]	7.2 ± 0.8 a	6.9 ± 1.0 a	6.3 ± 0.7 a
Arabinose [%]	4.9 ± 0.4 a	4.7 ± 0.5 a	3.8 ± 0.6 a
Mannose [%]	1.4 ± 0.2 a	1.3 ± 0.6 a	0.9 ± 0.3 a
β-glucan ** [%]	73.3 ± 1.7 a	72.3 ± 1.5 a	75.8 ± 2.0 a
Arabinoxylan ** [%]	6.9 ± 0.7 a	6.5 ± 1.1 a	5.2 ± 1.0 a
Galactomannan ** [%]	7.7 ± 0.9 a	7.4 ± 1.4 a	6.5 ± 0.9 a
Total sugar content [%]	87.9 ± 3.3 a	86.2 ± 4.0 a	87.5 ± 3.9 a
Protein [%]	6.5 ± 2.0 a	7.7 ± 2.1 a	7.9 ± 1.4 a
Molecular properties			
Weight-average molar mass—M_w_ [g/mol]	24,540	85,940 ***	1,714,770
Number-average molar mass—M_n_ [g/mol]	2140	24,380 ***	329,770
Dispersity (Ð = M_w_/M_n_)	11.5	3.5 ***	5.2

* BG-LMW—low-molar-mass hydrolyzed β-glucan; BG-MMW—medium-molar-mass β-glucan; BG-HMW—high-molar-mass β-glucan. **—AX content was calculated by taking the sum of arabinose and xylose content after acid hydrolysis and multiplying the result by a factor of 0.88. β-glucan content was calculated by multiplying the glucose content by a factor of 0.9. Galactomannan content was calculated by multiplying the sum of galactose and mannose content by a factor of 0.9. ***—From the major peak of the HPLC chromatograms. Mean values marked with the same letters in particular rows are not statistically significantly different at *p* < 0.05. nd—not determined.

**Table 2:** Water addition and dough yield of gluten-free bread without (control) and with addition of β-glucans.

Parameters	Control *	BG-LMW *		BG-MMW *		BG-HMW *	
	0%	1%	2%	1%	2%	1%	2%
Water addition [mL]	88.7 ± 1.5 a	89.4 ± 0.5 a,b	90.7 ± 0.9 b	93.3 ± 0.5 c	96.7 ± 0.0 d	100.0 ± 0.8 e	110.0 ± 0.5 f
Dough yield [%]	188.7 a	189.4 a,b	190.7 b	193.3 c	196.7 d	200.0 e	210.0 f

* Control—gluten-free bread without added β-glucan; BG-LMW—gluten-free bread with low-molar-mass β-glucan, hydrolyzed; BG-MMW—gluten-free bread with medium-molar-mass β-glucan; BG-HMW—gluten-free bread with high-molar-mass β-glucan. Mean values marked with the same letters in particular rows are not statistically significantly different at *p* < 0.05.

**Table 3:** Properties of gluten-free bread without (control) and with addition of β-glucan preparations.

Parameters	Control *	BG-LMW *		BG-MMW *		BG-HMW *	
	0%	1%	2%	1%	2%	1%	2%
Baking loss [%]	15.3 ± 0.8 a	15.3 ± 0.6 a	15.8 ± 1.6 a	14.7 ± 0.8 a	16.3 ± 1.8 a	15.0 ± 0.2 a	15.2 ± 1.0 a
Total baking Loss [%]	18.5 ± 0.7 a	19.8 ± 0.4 b	19.5 ± 1.4 a,b	19.9 ± 0.4 b	21.3 ± 0.5 c	19.4 ± 0.3 a	19.1 ± 0.7 a
Bread volume [cm^3^]	93.7 ± 3.1 a,b	101.7 ± 2.2 d	98.8 ± 1.1 c,d	100.7 ± 1.6 d	89.7 ± 3.2 a	101.2 ± 1.6 d	97.6 ± 1.7 b,c
Specific bread volume [cm^3^/100 g of flour]	294.6 ± 9.7 a	321.0 ± 6.9 b,c	314.0 ± 3.5 b	324.6 ± 5.2 c	294.0 ± 10.5 a	332.0 ± 5.3 c	341.6 ± 6.0 d

* Control—gluten-free bread without added β-glucan; BG-LMW—gluten-free bread with low-molar-mass β-glucan, hydrolyzed; BG-MMW—gluten-free bread with medium-molar-mass β-glucan; BG-HMW—gluten-free bread with high-molar-mass β-glucan. Mean values marked with the same letters in particular rows are not statistically significantly different at *p* < 0.05.

**Table 4:** Texture parameters of gluten-free bread without (control) and with share of β-glucans on the day of baking and after 1 day of storage.

Texture Parameter	Storage Time [Day]	Control *	BG-LMW *		BG-MMW *		BG-HMW *	
		0%	1%	2%	1%	2%	1%	2%
Hardness [N]	0	14.3 ± 1.3 c	8.1 ± 0.2 b	7.6 ± 0.3 b	8.8 ± 1.0 b	12.2 ± 1.1 c	8.3 ± 1.3 b	4.4 ± 0.4 a
	1	34.3 ± 2.8 g	18.8 ± 0.3 d	20.0 ± 0.1 e	17.0 ± 0.5 c	25.3 ± 3.1 f	14.8 ± 3.0 b	7.6 ± 0.1 a
Adhesiveness [cN·s]	0	−0.3 ± 0.2 a	0.0 ± 0.0 a	−0.2 ± 0.3 a	0.0 ± 0.0 a	−0.1 ± 0.1 a	−0.1 ± 0.2 a	0.0 ± 0.0 a
	1	−16.6 ± 0.9 c	−13.3 ± 2.3 b	−9.4 ± 3.2 b	−10.3 ± 2.0 b	−9.5 ± 2.1 b	−9.0 ± 1.7 b	−3.2 ± 0.8 a

* Control—gluten-free bread without added β-glucan; BG-LMW—gluten-free bread with low-molar-mass β-glucan, hydrolyzed; BG-MMW—gluten-free bread with medium-molar-mass β-glucan; BG-HMW—gluten-free bread with high-molar-mass β-glucan. Mean values marked with the same letters in particular rows are not statistically significantly different at *p* < 0.05.

**Table 5:** Moisture of the bread crumb in the central and peripheral parts of the model gluten-free bread without (control) and with the addition of β-glucans on the day of baking and after 1 day of storage.

Part of Bread Crumb	Storage Time [Day]	Control *	BG-LMW *		BG-MMW *		BG-HMW *	
		0%	1%	2%	1%	2%	1%	2%
Central	0	43.2 ± 0.6 a,B	45.7 ± 0.3 b,A	47.0 ± 0.3 c,A	46.4 ± 0.6 b,c,A	46.9 ± 0.3 c,A	48.7 ± 0.1 d,A	52.5 ± 0.3 e,B
	1	41.9 ± 0.8 a,A	46.1 ± 0.5 b,A	47.1 ± 0.4 c,A	46.9 ± 0.1 b,c,A	46.9 ± 0.3 b,c,A	48.3 ± 0.1 d,A	51.0 ± 0.4 e,A
Peripheral	0	42.7 ± 0.5 a,B	46.1 ± 0.4 b,B	46.9 ± 0.9 b,B	46.4 ± 0.3 b,A	47.1 ± 0.3 b,B	48.9 ± 0.4 c,B	51.1 ± 0.5 d,B
	1	40.6 ± 0.8 a,A	44.0 ± 0.0 b,A	44.9 ± 0.6 b,c,A	46.0 ± 0.1 d,A	45.9 ± 0.3 c,d,A	46.8 ± 0.4 d,A	48.1 ± 0.3 e,A

* Control—gluten-free bread without added β-glucan; BG-LMW—gluten-free bread with low-molar-mass β-glucan, hydrolyzed; BG-MMW—gluten-free bread with medium-molar-mass β-glucan; BG-HMW—gluten-free bread with high-molar-mass β-glucan. Mean values marked with the same small letters in particular rows are not statistically significantly different at *p* < 0.05. For central and peripheral parts of crumb separately, mean values marked with the same capital letters in particular columns are not statistically significantly different at *p* < 0.05.

## Data Availability

Data are contained within the article and Supplementary Materials.

## Supplementary Materials

The following supporting information can be downloaded at: https://www.mdpi.com/article/10.3390/molecules29194579/s1, Table S1: Analysis of variance (two-way ANOVA). The effect of molar mass of β-glucan and β-glucan share on bread crumb moisture of model gluten-free bread on the day of baking and after 1 day of storage.; Table S2: Analysis of variance (two-way ANOVA). The effect of molar mass of β-glucan, β-glucan share, storage time on bread crumb moisture in the central and peripheral parts of model gluten-free bread.; Table S3: Analysis of variance (two-way ANOVA). The effect of molar mass of β-glucan and β-glucan share on properties of model gluten-free bread.
