# Supplementary material for: The Influence of Oat β-Glucans of Different Molar Mass on the Properties of Gluten-Free Bread
Source: Molecules. 2024 Sep 26;29(19):4579. doi: 10.3390/molecules29194579 (PMC11478284; doi:10.3390/molecules29194579)
Supplement: Supplementary file 1 [file molecules-29-04579-s001.zip › molecules-3153358-supplementary.pdf]

**Supplementary Table S1.** Analysis of variance (two-way ANOVA). The effect of molar mass of β-glucan and β-glucan share on bread crumb moisture of model gluten-free bread on the day of baking and after 1 day of storage.

| | **Bread crumb moisture** | |
|---|---|---|
| | **F** | **p** |
| β-glucan type (molar mass) | 257.7 | * |
| β-glucan share | 91.0 | * |
| Storage time | 81.7 | * |
| Part of bread | 54.2 | * |
| Storage time*Part of bread | 30.9 | * |

ns — not significant at p≤0.05.
* — significant at p≤0.05.

**Supplementary Table S2.** Analysis of variance (two-way ANOVA). The effect of molar mass of β-glucan, β-glucan share, storage time on bread crumb moisture in the central and peripheral parts of model gluten-free bread.

| **β-glucan type (molar mass)** | **β-glucan share [%]** | **Storage time [day]** | **Part of bread** | **Bread crumb moisture [%]** |
|---|---|---|---|---|
| Control | | | | 42.1±1.2a |
| NM | | | | 46.6±0.5b |
| LMW | | | | 46.0±1.1b |
| HMW | | | | 49.4±1.9c |
| | 0 | | | 42.1±1.2a |
| | 1 | | | 46.7±1.4b |
| | 2 | | | 48.0±2.3c |
| | | 0 | | 47.1±2.6b |
| | | 1 | | 46.0±2.6a |
| | | | Central part | 47.0±2.7b |
| | | | Peripheral part | 46.1±2.6a |
| | | 0 | Central part | 47.2±2.8b |
| | | 0 | Peripheral part | 47.0±2.5b |
| | | 1 | Central part | 46.9±2.6b |
| | | 1 | Peripheral part | 45.2±2.3a |

Average ± SD; values within the column section denoted with the same superscript are not statistically different at p≤0.05.

**Supplementary Table S3.** Analysis of variance (two-way ANOVA). The effect of molar mass of β-glucan and β-glucan share on properties of model gluten-free bread.

| | WA | | DY | | BL | | TBL | | BV | | SBV | | Hardness 0d | | Adh 0d | | CM 0d | | PM 0d | | Hardness 1d | | Adh 1d | | CM 1d | | PM 1d | |
|---|---|---|---|---|---|---|---|---|---|---|---|---|---|---|---|---|---|---|---|---|---|---|---|---|---|---|---|---|
| | F | p | F | p | F | p | F | p | F | p | F | p | F | p | F | p | F | p | F | p | F | p | F | p | F | p | F | p |
| Molar mass of β-glucan | 699 | * | 699 | * | 0,4 | ns | 7,4 | * | 9,6 | * | 25,2 | * | 22,2 | * | 0,1 | ns | 138,8 | * | 54,0 | * | 29,6 | * | 7,2 | * | 54,7 | * | 46,2 | * |
| β-glucan share | 167 | * | 167 | * | 2,6 | ns | 0,7 | ns | 33,8 | * | 8,3 | * | 0,3 | ns | 0,4 | ns | 66,2 | * | 16,4 | * | 0,5 | ns | 9,1 | * | 23,0 | * | 6,9 | * |
| Molar mass of β-glucan * β-glucan share | 90 | * | 90 | * | 0,9 | ns | 3,9 | * | 6,7 | * | 12,8 | * | 16,1 | * | 1,0 | ns | 18,0 | * | 2,4 | ns | 15,6 | * | 1,5 | ns | 8,7 | * | 2,8 | ns |

ns — not significant at $p \le 0.05$.
* — significant at $p \le 0.05$.

WA – water addition; DY – dough yield; BL – baking loss; TBL – total baking loss; BV – bread volume; SBV – specific bread volume; Adh – adhesiveness; CM - moisture of the crumb in the central and peripheral (CP) part. 0d – day of baking; 1d – after 1 day of storage.
